# Why and How to Approach User Experience in Safety-Critical Domains: The Example of Health Care

**DOI:** 10.1177/0018720819887575

**Published:** 2020-01-08

**Authors:** Tobias Grundgeiger, Jörn Hurtienne, Oliver Happel

**Affiliations:** Julius-Maximilians-Universität Würzburg, Würzburg, Germany; University Hospital Würzburg, Würzburg, Germany

**Keywords:** activity theory, concepts of interaction, safety-critical domains, embodiment, eudaimonia, interaction as experience, user experience

## Abstract

**Objective:**

To highlight the importance of the personal experience of users who interact with technology in safety-critical domains and summarize three interaction concepts and the associated theories that provide the means for addressing user experience.

**Background:**

In health care, the dominant concepts of interaction are based on theories arising from classic cognitive psychology. These concepts focus mainly on safety and efficiency, with too little consideration being given to user experience.

**Method:**

Users in complex socio-technical and safety-critical domains such as health care interact with many technological devices. Enhancing the user experience could improve the design of technology, enhance the well-being of staff, and contribute to modern safety management. We summarize concepts of “interaction” based on modern theories of human–computer interaction, which include the personal experience of users as an important construct.

**Results and Conclusion:**

Activity theory, embodiment, and interaction as experience provide a theoretical foundation for considering user experience in safety-critical domains. Using an example from anesthesiology, we demonstrate how each theory provides a unique but complementary view on experience. Finally, the methodological possibilities for considering personal experience in design and evaluations vary among the theories.

**Application:**

Considering user experience in health care and potentially other safety-critical domains can provide an additional means of optimizing interaction with technology, contributing to the well-being of staff, and improving safety.

## Introduction

In a well-known textbook on human factors (Lee, Wickens, Liu, & Boyle, 2017), usability is described in terms of how intuitive and easy it is to interact with an (often technological) artifact. Common aspects of this interaction include efficiency, effectiveness, errors, and satisfaction (e.g., Nielsen, 1993). However, the exact aspects of this interaction can differ widely. Hornbæk and Oulasvirta (2017) described how the view on interaction depends on the theoretical concept that a researcher or practitioner implicitly or explicitly adopts. The theoretical concept determines what is considered more or less important in the interaction, the key constructs, and what is viewed as “good” or “successful” interaction. For example, considering interaction as the transmission of information, a sender sends a message over a noisy channel (e.g., Lee et al., 2017). The key constructs are the sending of the messages (in bits) between a sender and a receiver. Good interaction is indicated by a maximum throughput of information (i.e., a fast interaction). A contrasting example is the concept of interaction as an experience (e.g., Hassenzahl, Platz, Burmester, & Lehner, 2000). Interaction is viewed as an ongoing experience that not only refers to the very moment one is interacting with technology but also considers the time before and after the interaction. It includes the key concepts of expectation, non-utilitarian quality, and emotion (e.g., the joy of use). Good interaction is indicated, for example, by the satisfaction of psychological needs (Hassenzahl, 2010).

In the present article, we argue that a one-sided view of interaction in health care, which focuses on efficacy and the absence of errors, has resulted in the neglect of user experience (UX) when interacting with technology. We summarize three views on interaction that emphasize UX, argue why they warrant consideration in health care, and explain how these views recommend addressing UX. Although our examples center on health care, we suggest that UX is equally important in other safety-critical domains. The general applicability of the theories, on which the three views on interaction are based, enable researchers and designers to consider UX in any safety-critical domain. Finally, we would like to note that we suggest including additionally UX considerations in the design and evaluation of technology, rather than replacing classic usability metrics.

## Interaction in Safety-Critical Domains

Socio-technical, safety-critical domains such as aviation, plant control, or health care involve differences (Durso & Drews, 2010) but also similarities: Operators receive extensive training, frequently work in teams, and encounter many technological devices in the workplace; a situation can change quickly; the processes involved cannot be paused or stopped; and operators work toward specific goals. For example, the induction of general anesthesia has the aim of (a) rapidly, safely, and pleasantly producing anesthesia in the patient while (b) maintaining hemodynamic stability and ventilation (King & Weavind, 2017). The anesthesiologist and the anesthetic nurse use several drugs and tools to induce this target state. The anesthesiologist, for example, uses a device to obtain a view of the larynx along with the vocal cords (so-called laryngoscopy) to be able to insert a tube in the windpipe (so-called endotracheal intubation) and secure the airway of the patient for mechanical ventilation. The effect of these technological and pharmacological interventions is continuous (i.e., they cannot be immediately stopped), while the status of the patient may change quickly.

**Figure 1.**
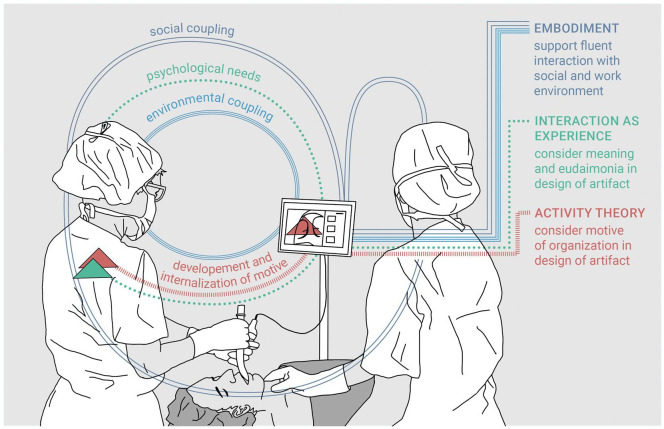
Illustration of the main points of the three theories in relation to user experience. The anesthesiologist is performing an endotracheal intubation with a video laryngoscope. The anesthetic nurse is displacing the larynx using a manual maneuver to enable a better view of the vocal cords. Social and environmental coupling can support the experience of a fluent interaction (embodiment): The anesthesiologist does not need to provide verbal commands to the anesthetic nurse to get a better view as would be necessary in the case of direct laryngoscopy (social coupling), and the device enables the anesthesiologist to focus on the intended task (environmental coupling). The interaction can satisfy the psychological needs of the user (interaction as experience): Video laryngoscopes enable easier endotracheal intubation for difficult airways and therefore can provide a feeling of competence. The motives of the organization are part of the design and affect the needs and motives of the user (activity theory): In the case of aspiration of stomach contents, the anesthetic nurse can immediately provide assistance. For detailed descriptions of the examples, see the discussion section.

Research and design regarding human–system interaction in safety-critical domains focus on interaction concepts related to safety and efficiency (Norros, 2014). The dominant interaction concepts are based on “classic” or “first-wave” human–computer interaction theories (for an overview, see Bødker, 2006; Rogers, 2012). In short, classic theories refer to basic and applied cognitive psychology theories of information processing and modeling (Rogers, 2012). For example, the above-mentioned induction of general anesthesia in a patient has been considered to be a supervisory control task (e.g., Drews, Syroid, Agutter, Strayer, & Westenskow, 2006; Gaba, Howard, & Small, 1995). The interaction relates to achieving a target state (i.e., the induction of general anesthesia) and a good interaction is indicated by producing this target state rapidly, safely, and pleasantly in the patient. In relation to the tools used during the induction of general anesthesia, research showed that different devices for laryngoscopy and endotracheal intubation result in different types of performance. Traditional devices include a blade with a light at the tip, which is used to establish a direct view of the vocal cords. Video-based devices have a camera along with a light attached at the tip of the blade, which is inserted in the patient’s mouth and streams the resulting video on a display monitor (see Figure 1). The first video-based laryngoscopes were developed to enable a better view and faster tracheal intubation for difficult airways (e.g., Cooper, 2003), and a review showed that video-based laryngoscopy outperforms direct laryngoscopy in relation to efficiency (Griesdale, Liu, McKinney, & Choi, 2012).

“Modern” or “second and third wave” concepts include theories and frameworks from diverse traditions (Rogers, 2012). A brief summary of modern interaction concepts based on Hornbæk and Oulasvirta (2017) is given in Table 1. Modern and contemporary theoretical concepts describe good interaction in terms of, for instance, resources for and in support of fluent participation in the world (i.e., users are not aware that they are using technology to pursue an intention, with technology only having a presence if something unexpected happens; interaction as embodiment). Modern and contemporary concepts are not generally utilized in health care and other safety-critical domains (Norros, 2014), and we see a particular lack in relation to UX. In health care, for example, the above-cited meta review on the evaluation of video-based laryngoscopy versus direct laryngoscopy evaluated only the view on the vocal cords, the number of intubation attempts, and the time needed for intubation (Griesdale et al., 2012). Furthermore, evaluations of anesthesia machines (Jiang, Liu, Feng, Gao, & Zhang, 2018; Spaeth, Schweizer, Schmutz, Buerkle, & Schumann, 2017), a review on touchless interaction in interventional radiology and surgery (Mewes, Hensen, Wacker, & Hansen, 2017), and a review on augmented reality (Dey, Billinghurst, Lindeman, & Swan, 2018) have shown that some evaluations have exclusively focused on precision, errors, and time-saving.

**Table 1:** Summary of Interaction Concepts After Hornbæk and Oulasvirta (2017) That Are Based on Modern Human–Computer Interaction Theories (Rogers, 2012). In the Far-Right Column, We Suggest Design and Evaluation Methods to Address User Experience

Interaction as . . .	Interaction Is Viewed as . . .	Good Interaction Is Indicated by . . .	Possible Design and Evaluation Methods
Tool use	a human who uses a tool to perceive or manipulate the world.	a useful and transparent tool that amplifies human capabilities.	Qualitative: inquiring about the feeling and subjective experience of interaction and projecting tool use onto future work (e.g., Savioja, Liinasuo, & Koskinen, 2014); studying habits of action that relate to the motives of users and organizations
Embodiment	being with our bodies in a physical and social world and acting in specific situations.	the possibility of a fluent participation in the physical and social worlds.	Qualitative: observation of and interviews about breakdowns of fluent interaction; focus on intention of users
Experience	an ongoing stream of experiences and feelings.	the satisfaction of psychological needs.	Qualitative: interviews focusing on psychological need satisfaction; Quantitative: psychological need scales (e.g., Hassenzahl, Wiklund-Engblom, Bengs, Hägglund, & Diefenbach, 2015)

To the best of our knowledge, in health care and other safety-critical domains, UX has received limited attention in practice and research (however, see McCarthy & Wright, 2005; Savioja, Liinasuo, & Koskinen, 2014). In the aforementioned textbook on human factors, Lee et al. (2017) describe satisfaction as a human factors goal that does not need to be emphasized in high-risk domains and in workplaces. Similarly, Grudin (2017) noted in another textbook on human–computer interaction in reference to safety-critical domains that “error reduction is critical, performance enhancement is good, and other goals are less important” (p. 92), and the U.S. Food and Drug Administration (2016) “is primarily concerned that devices are safe and effective for the intended users, uses, and use environments” (p. 1f). Finally, it has been questioned whether UX in safety-critical domains should be considered at all or whether it is even irrelevant (Mentler & Herczeg, 2016).

## Why UX Should be Considered

In our view, there are several reasons why UX should be considered when interacting with technology in safety-critical domains. First, interaction with technology always has an associated UX. This UX exists regardless of whether or not it is actually addressed by researchers and designers. For example, a manufacturer of medical equipment commented to the current authors that a non-functional, visual feature of a new product had a stronger positive effect than new functionalities, since the visual feature indicated the latest product. As a further example, when operating medical equipment, medical staff, while never intending to make mistakes, may have to live with the experience of having caused patient harm after an incident (Wu & Steckelberg, 2012).

Considering that each interaction has an associated UX, it is not too surprising that UX is included in the ISO 9241-210 (2019). Another, less discussed ISO, 9241-2 (1992) “Guidance on task requirements,” stresses the well-being of individual users by applying knowledge about ergonomics and psychology at work. Considering and improving the UX is part of one of the fundamental aims of standards on human factors and human–computer interaction.

Second, UX can help with the design of better tools. As we describe in detail below, Savioja et al. (2014) argued, that during the development of technology, assessing the potential of a future work tool depends on domain experts “gut feelings” or sentiments that are the result of projecting tool use onto future work. Considering UX, therefore, can make a direct contribution to the design of tools that are more likely to be usable and accepted.

Third, UX can support contemporary safety management. Hollnagel (2014) describes safety as the state where few errors occur (Safety I). In the Safety I view, errors occur because of the malfunctioning of equipment, poor usability, the wrong procedures being followed, the right procedures being improperly followed, and so on. To achieve safety, one must identify hazards and liabilities, fix the associated problems by eliminating causes or introducing barriers, and try to keep errors to a minimum (Braithwaite, Wears, & Hollnagel, 2015). The Safety I approach is necessary and valuable, and the classic cognitive approaches to usability can provide valuable methods by which to improve Safety I. However, modern socio-technical systems are often too complex and varied to identify single or multiple hazards and liabilities. Hollnagel, Wears, and Braithwaite (2015) make the point that “things do not go right because people behave as they are supposed to, but because people can and do adjust what they do to match the conditions of work” (p. 4).

Hollnagel and colleagues extend the Safety I approach with the Safety II approach, which aims to ensure that as many things as possible go right and that research can learn from success rather than failure. In the Safety II approach, humans are not considered to be a liability or a hazard but a resource that enables the socio-technical system to adapt to varying conditions (Hollnagel et al., 2015). Based on the approach of Safety II risk management, staff need to be supported to deal with varying conditions. We argue that recent advances in UX can contribute to Safety II by considering eudaimonia—seeking to use and develop one’s potential (Huta & Ryan, 2010; Mekler & Hornbæk, 2016)—and eudaimonia-related needs, such as competence and autonomy, in the interaction design of tools. In health care, for example, staff have been described as being the ones to “finish the design” or “close the gap” if a particular system or technology fails to support the user (Cook, Render, & Woods, 2000). Staff who experience competence when interacting with technology may be better equipped to fill this gap, to keep a process going, to reestablish safety, or to enable safety in the first place.

Fourth, the introduction of technology in organizations frequently faces many barriers (Harrison, Gajic, Pickering, & Herasevich, 2016; Yang, Zimmerman, Steinfeld, Carey, & Antaki, 2016), with the lack of appropriate interaction design having been specifically pointed out as the main challenge (Sittig et al., 2008). Neglecting UX may contribute to the non-acceptance of technology. In relation to electronic patient records, McCarthy and Wright (2005) report that nurses preferred a manual record to an electronic record despite performance benefits. The reasons were, for example, anxiety about technological competence, a lack of trust in data quality, or the feeling that the electronic system will take them away from the patient. In relation to clinical decision support tools, Valenta et al. (2010) reported that physicians strongly agreed with the statement that decision support tools intrude professional autonomy and undermine the competence of physicians. Similarly, Yang et al. (2016) studied barriers to the adoption of clinical decision support tools and stated that a decision support tool should have the “explicit goal of helping clinicians feel they are doing better work, and not necessarily automating the part of work that makes them feel like an expert” (p. 4484). These examples highlight the fact that considering UX, in the form, for example, of psychological needs, seems to play a role in successful technology implementation, with missing UX possibly contributing to the non-acceptance of technology.

## Three Approaches to Ux

In this section, we briefly introduce three concepts of interaction (Table 1), which are based on modern theories, and the role of UX within these theories. Furthermore, we highlight how each theory can provide the means for addressing the above reasons in the study of UX. We have illustrated the main points of each theory in Figure 1. We choose these three views because they have a strong historical and theoretical background, they have been related to UX, and we could see merits in applying these theories to the design of technology in health care and potentially other safety-critical domains. Note that there are other views on UX (Bargas-Avila & Hornbæk, 2011; Hornbæk & Hertzum, 2017; Obrist et al., 2012) and concepts of interaction in general (Hornbæk & Oulasvirta, 2017) that we did not consider.

### Activity Theory: Interaction as Tool Use

Activity theory considers technology as a tool that people use to complete activities. Tools mediate the relationship between the actor (i.e., the user) and the object (i.e., the intended end state of the system) during activities and both the user and the object are affected by the tools (Engeström, 2014). The object may be affected in terms of a changed state whereas the actor may be affected by learning a new function of a tool or a new way of practice (Norros, 2014).

To understand the mediating role of tools in relation to UX in safety-critical domains, Savioja et al. (2014) introduced the concept of *instrumental experience*, which exploits the notion that UX can reflect information about the status of an activity. Kaptelinin and Nardi (2012) pointed out that the subjective experience of the user is closely tied to the activity and that users’ feelings act as indicators of activity status (i.e., of how well they are doing). Therefore, a “good” UX is not only tied to a single element in the interaction with a tool, or a moment of pleasure when interacting with technology, but to a successful activity. The concept of instrumental experience, therefore, aims to address feelings of effectiveness and efficiency as well as the state of being effective and efficient. Savioja et al. (2014) investigated feelings of effectiveness and efficiency by means of a questionnaire addressing the role of three functions of a tool in an activity (instrumental, psychological, and communicative; see also Savioja & Norros, 2013) in the evaluation of different power plant control room designs.

Savioja et al. (2014) argued that considering UX is particularly important when developing new tools because only domain experts have the necessary experience to judge the potential of new tools. However, expert judgment might prove difficult to verbalize and be manifested in a “gut feeling” about whether the prototype or the tool will be suitable for the job. Therefore, UX may be a valuable concept in safety-critical domains in the development of new technology and interaction paradigms.

### Embodied Cognition: Interaction as Embodiment

Interaction as embodiment highlights the intentionality of the user, the coupling of the user and the environment (e.g., devices), and the context of the interaction (Dourish, 2004). A user with a physical body acts intentionally in a physical and social environment. These actions result in specific experiences—the “felt” or “lived” experiences—which are central to embodiment. There are many facets of embodiment (Hornecker, Marshall, & Hurtienne, 2017), and we see merits in considering experience from a first-person viewpoint (i.e., the view of the actual operator) when designing interactions in specific safety-critical domains. We therefore focus on the interaction of the physical body in the environment (rather than the effect of the body on cognition) and the situatedness and social aspects of action (rather than the cognitive structures that might be involved in interaction with technology).

With particular emphasis on the personal experience of the user, McCarthy and Wright (2005) suggested “putting ‘felt-life’ at the centre of human-computer interaction” as well as focusing on both the cognitive aspects (e.g., efficiency, errors) and the sensual and emotional aspects (e.g., experience, feelings, engagement). McCarthy and Wright (2005) argue that the sensual and emotional aspects add information about trust, closeness, self-reflexivity, or anxiety. As indicated above, McCarthy and Wright (2005) refer to a study of a hospital information system to illustrate how a qualitative approach helped to understand the personal viewpoint of users (i.e., nurses), their values, and goals (McCarthy & O’Connor, 1999). The qualitative approach facilitated an explanation of why the hospital information system was not well perceived by the nurses and why nurses resisted using the system. Similar to the instrumental experience approach of Savioja et al. (2014), using a felt-life framework and qualitative methods to understand the personal view of the users, their values, and their goals may also be helpful to the design of technology.

Specifically designing for an embodied being-in-the-world, van Dijk and Hummels (2017) emphasize a skillful coupling of the user with (a) the environment and (b) social coordination when acting in the world. These two aspects are highly relevant in safety-critical domains when expert users employ technological devices and work in teams. Technological devices only produce the desired output through interaction with a skillful person. A skillful action is characterized by a parallel action-perception coupling rather than a linear perception-processing-action procedure (van Dijk & Hummels, 2017). From this point of view, it is critically important to consider the felt experience of the user when designing tools that enable resources and support the fluent interaction with the work environment. Technological devices also have an effect on social coordination; therefore, they impact not only the experience and actions of the user but also those of other individuals (Grundgeiger et al., 2019).

### Experience Design: Interaction as Experience

The concept of the fulfillment of psychological needs and the associated positive emotions was an early concept for UX (Hassenzahl et al., 2000). A distinction is made between the pragmatic and the hedonic qualities of a product (Hassenzahl, 2010). Pragmatic qualities are related to instrumental and task-oriented attributes; hedonic qualities, in contrast, are related to excitement, enjoyment, pleasure, or novelty.

More recently, the concept of eudaimonia—the idea of striving for the realization of one’s personal potential (Huta & Ryan, 2010; Mekler & Hornbæk, 2016)—has been introduced to human–computer interaction in relation to interaction with technology. Unlike hedonia, eudaimonia does not place an emphasis on immediate pleasure as part of UX but highlights value and meaningfulness (Botella et al., 2012; Desmet & Hassenzahl, 2012). In consumer interactions with technology, eudaimonia has been empirically related to the human need for competence and self-actualization as well as with the pragmatic quality of a product (Mekler & Hornbæk, 2016). Beyond the interaction of an individual with technology, Botella et al. (2012) added a discussion on the social and interpersonal level of interaction, along with the objective of facilitating information-sharing, mutual care, and the flourishing of community life.

Supporting a meaningful UX when interacting with technology provides the means for fulfilling the criteria of ISO 9241-2 (1992), which includes the well-being of individual users at work. In the context of consumer products, well-being is often aligned with hedonic experiences such as enjoyment and pleasure which may not be achievable in safety-critical domains. Well-being in relation to eudaimonia refers to finding virtue (personal expressiveness, the actualization of human potential, or activities that are congruent with a user’s values) by satisfying the three psychological needs of autonomy, competence, and relatedness (Ryan & Deci, 2001). Considering meaning and eudaimonia in the interaction with technology is a recent trend in consumer human–computer interaction (for a summary, see Mekler & Hornbæk, 2019), and in our view, a promising way of supporting the well-being of staff in safety-critical domains. Beyond the personal benefits for staff, as outlined above, we suggest that an interaction with technology, which can contribute, for example, to the feeling of autonomy and competence can serve as a means of supporting a Safety II approach. That is, staff may be better enabled to deal adaptively with varying conditions to establish safety (Hollnagel et al., 2015). In addition, considering the psychological needs of autonomy and competence in the design may also contribute to technology acceptance. Finally, the objectives at the social and interpersonal level (information-sharing, mutual care, and growing a community life) may also have matching aspects in safety-critical domains. The concept of Crew Resource Management (Helmreich, Merritt, & Wilhelm, 1999), for example, emphasizes the information-sharing and working culture aspects of teamwork in safety-critical domains, which can also be facilitated by computing technology.

## Discussion

The theories behind the three views on interaction approach UX from different perspectives. The theories have different foci and make distinct contributions to the research agenda in investigating UX with technology in safety-critical domains. However, all three theories consider UX and have overlaps in their historic roots and involved concepts (Hassenzahl, 2010; Kaptelinin & Nardi, 2012; Norros, 2014). From a practical perspective, the theories should be considered as complementary with different foci on UX and different methodological suggestions for addressing UX.

Activity theory emphasizes the agency of individuals, teams and organizations (Kaptelinin & Nardi, 2012). Therefore, viewing interaction as tool use also underlines the importance of considering the motives/needs of larger organizational units when investigating UX in safety-critical domains. The approach of Savioja et al. (2014) to UX focuses on operators’ activities, which are likely to be influenced by the motives or objectives of larger organizational units. In health care, organizational motives include, for example, patient safety, or economic efficiency. The concept of instrumental experience proposed by Savioja et al. (2014), therefore, focuses very much on safety and efficiency; consideration is given, however, to the value of UX in achieving these aims. Furthermore, the design of technology should explicitly consider the motives and needs of the organization because the tools always affect the object (i.e., the state of the system) and the actor (i.e., the user). For example, in his clinical practice, author O. H. uses video-based laryngoscopy for so-called rapid sequence induction. Rapid sequence inductions are used for the induction of general anesthesia in the case of patients with a high risk of regurgitation and pulmonary aspiration of stomach contents. With a video-based laryngoscope, the whole anesthesia team can see immediately on the display monitor of the video laryngoscope whether the patient is regurgitating (see Figure 1). Behavior such as using a tool in anticipation of possible problems can be considered as a habit that indicates what an individual or community considers to be worthy and valuable (Norros, 2014). For the design of new technology, studying and analyzing such habits of action can provide insights into what users or organizations value (Norros, 2014). In turn, these insights can be used to inform design. Technology that mediates the values of an organization make it more likely that users internalize these values.

Methodologically, the approach of Savioja et al. (2014) provides an example of the use of a questionnaire to consider instrumental experience during the design and evaluation of new technology in the context of nuclear power plant control. The questions specifically address the feelings of the users, for example, by starting with “In my experience . . . ” or “Learning to use the . . . system feels effortless.” Karvonen, Koskinen, and Haggrén (2012) describe a four-step procedure to elicit UX goals for future technological concepts, starting with users’ activity as a theoretical underpinning (Savioja & Norros, 2013). Karvonen and colleagues also describe an evaluation method for a prototype of the technological concepts, including a comparison of two designs, a questionnaire, and interviews (Karvonen, Koskinen, Tokkonen, & Hakulinen, 2014).

The theoretical view of interaction as experience may be viewed as complementary to activity theory given its focus on an individual’s psychological need fulfillment as a feature of interaction with technology. Future research in safety-critical domains needs to investigate the relationship between experiences with technology, on the one hand, and eudaimonic motives and specific needs, such as those for competence, self-actualization, and security, on the other. For example, video-based laryngoscopy enables a senior anesthesiologist to monitor what a junior anesthesiologist sees and does during laryngoscopy and the placement of the endotracheal tube (Berkow, Morey, & Urdaneta, 2018). The tool and the resulting situation provide the senior anesthesiologist with the reassurance that he or she knows what the junior anesthesiologist is doing and provides the junior anesthesiologist the opportunity to experience competence by conducting the laryngoscopy and endotracheal intubation. Need-related concepts have recently also been the focus of frameworks (Lu & Roto, 2015) and empirical work (Harbich & Hassenzahl, 2017) in general work settings. These insights may help to inform design methods on need-related UX in safety-critical domains. Qualitative methods may be useful for design processes. For example, focusing on psychological need satisfaction such as autonomy and competence in the design of decision support systems may improve the acceptance of technology. Quantitative approaches may be useful for a summative evaluation of prototypes. For example, need satisfaction scales (e.g., Hassenzahl, Wiklund-Engblom, Bengs, Hägglund, & Diefenbach, 2015; Sheldon, Elliot, Kim, & Kasser, 2001) could be used to compare the decision support systems that have been specifically designed to satisfy psychological needs versus “standard” designs.

The phenomenological approach of interaction as embodiment can provide a detailed snapshot of how an operator experiences interaction with a device and what specific situational and social aspects contribute to this experience. Future research could aim to collect experience “categories” for safety-critical domains—categories of repeatedly occurring (mainly positive) qualities and their necessary, or optional, attributes (Zeiner et al., 2018). Experience categories could then be used in the design of future devices. Another approach is designing for an embodied being-in-the-world by concentrating on the integration of technology into the so-called socio-sensorimotor loop of the operator. van Dijk and Hummels (2017; van Dijk, van der Lugt, & Hummels, 2014) suggested that there are different entry-points into the loop. For example, entry points enable the user to *sense* new information in the environment or create new opportunities and possibilities to *act*. As described above, video-based laryngoscopy allows the whole anesthesia team to view the vocal cords during endotracheal intubation. The anesthetic nurse can see (i.e., sense) a view of the vocal cords of the patient and anticipate whether and how to support the anesthesiologist, for example, by displacing the larynx using a manual maneuver to provide a better view of the vocal cords (see Figure 1). Without the video view, the maneuver has been described as tedious (Hindmarsh & Pilnick, 2007). The nurse could also provide immediate support to the anesthesiologist if the patient regurgitates gastric contents during the procedure. Video-based laryngoscopy, therefore, provides ways for supporting the above-described skillful coupling of the user with (a) the environment and (b) social coordination when acting in the world. Methodological, qualitative research such as observations that underline how the environment can support the pursuit of the user and note breakdowns in current practice could provide insights for design purposes.

We suggested three theoretical interaction concepts that seem appropriate for addressing UX in health care and possibly in other safety-critical domains. One limitation of the article is that we did not conduct a formal review of the literature to identify which approaches and theories relevant to UX might be the most suitable to study UX in safety-critical domains. Furthermore, we only selectively presented empirical studies from the field of UX research. Finally, open questions remain for future research. For example, based on the literature and the methodological traditions of the interaction concepts, we suggested methods by which to consider UX in design and to conduct UX research. However, the methods might need to be tailored to safety-critical domains, while UX research is required to provide data to determine, for example, the relative importance of psychological needs in the interaction with technology in different safety-critical domains. Furthermore, the development of UX over time and the effect of expertise on UX are worthwhile topics for future research. Activity theory as part of instrumental experience in the design of tools might be suitable during prototyping; however, activity theory also includes the technology-mediated development of motives, meaning that a longer period may be necessary. The experience design literature stresses the temporal aspects in UX (Hassenzahl, 2010; McCarthy & Wright, 2004). Questionnaires using psychological need scales could be used to evaluate prototypes (but such variables also change over time, see Harbich & Hassenzahl, 2017); however, empirical work on well-being has shown that eudaimonically motivated activity has a delayed (i.e., months) positive effect on well-being (Huta & Ryan, 2010).

## Conclusion

The dominant interaction concepts in safety-critical domains do not explicitly include UX. However, whether designed or not, operators in safety-critical domains such as health care do experience interaction with technology, and we have argued that considering UX could improve the design of technology, enhance the well-being of staff, and contribute to modern safety management. Concepts of interaction based on modern human–computer interaction theories and research on UX provide promising starting points for much-needed research and design agendas in the case of UX in safety-critical domains.

## Key Points

- Within health care and other safety-critical domains, user experience has received limited attention in practice and research.
- Users in safety-critical domains such as health care experience interaction with technology, and we argue that considering experience could improve the design of technology, improve the well-being of staff, and contribute to modern safety management.
- Interaction concepts which are based on modern theories (activity theory, embodiment, and interaction as experience) from different research traditions provide a way for addressing user experience in safety-critical domains, such that the well-being of staff and patient safety are improved.

## References

[bibr1-0018720819887575] Bargas-Avila J. A. Hornbæk K. (2011, May 7–12). Old wine in new bottles or novel challenges: A critical analysis of empirical studies of user experience. Paper presented at the Proceedings of the SIGCHI Conference on Human Factors in Computing Systems, Vancouver, British Columbia, Canada.

[bibr2-0018720819887575] Berkow L. C. Morey T. E. Urdaneta F. (2018). The technology of video laryngoscopy. Anesthesia and Analgesia, 126, 1527–1534. doi:10.1213/ANE.0000000000002490 28961559

[bibr3-0018720819887575] Bødker S. (2006, October 14–18). When second wave HCI meets third wave challenges. Paper presented at the 4th Nordic Conference on Human-Computer Interaction Changing Roles, Oslo, Norway.

[bibr4-0018720819887575] Botella C. Riva G. Gaggioli A. Wiederhold B. K. Alcaniz M. Banos R. M. (2012). The present and future of positive technologies. Cyberpsychology, Behavior, and Social Networking, 15, 78–84. doi:10.1089/cyber.2011.0140 22149078

[bibr5-0018720819887575] Braithwaite J. Wears R. L. Hollnagel E. (2015). Resilient health care: Turning patient safety on its head†. International Journal for Quality in Health Care, 27, 418–420. doi:10.1093/intqhc/mzv063 26294709

[bibr6-0018720819887575] Cook R. I. Render M. Woods D. D. (2000). Gaps in the continuity of care and progress on patient safety. British Medical Journal, 320, 791–794. doi:10.1136/bmj.320.7237.791 10720370 PMC1117777

[bibr7-0018720819887575] Cooper R. M. (2003). Use of a new videolaryngoscope (GlideScope®) in the management of a difficult airway. Canadian Journal of Anesthesia, 50, 611–613. doi:10.1007/bf03018651 12826557

[bibr8-0018720819887575] Desmet P. Hassenzahl M. (2012). Towards happiness: Possibility-driven design. In Zacarias M. de Oliveira J. V. (Eds.), Human-computer interaction: The agency perspective (pp. 3–27). Berlin, Germany: Springer Berlin Heidelberg.

[bibr9-0018720819887575] Dey A. Billinghurst M. Lindeman R. W. Swan J. E. (2018). A systematic review of 10 years of augmented reality usability studies: 2005 to 2014. Frontiers in Robotics and AI, 5, Article 37. doi:10.3389/frobt.2018.00037 PMC7805955 33500923

[bibr10-0018720819887575] Dourish P. (2004). Where the action is: The foundations of embodied interaction. Cambridge, MA: The MIT Press.

[bibr11-0018720819887575] Drews F. A. Syroid N. Agutter J. Strayer D. L. Westenskow D. R. (2006). Drug delivery as control task: Improving performance in a common anesthetic task. Human Factors, 48, 85–94. doi:10.1518/001872006776412216 16696259

[bibr12-0018720819887575] Durso F. T. Drews F. A. (2010). Health care, aviation, and ecosystems: A socio-natural systems perspective. Current Directions in Psychological Science, 19, 71–75. doi:10.1177/0963721410364728

[bibr13-0018720819887575] Engeström Y. (2014). Learning by expanding: An activity—Theoretical approach to developmental research. Cambridge, UK: Cambridge University Press.

[bibr14-0018720819887575] Gaba D. M. Howard S. K. Small S. D. (1995). Situation awareness in anesthesiology. Human Factors, 37, 20–31. doi:10.1518/001872095779049435 7790008

[bibr15-0018720819887575] Griesdale D. E. G. Liu D. McKinney J. Choi P. T. (2012). Glidescope® video-laryngoscopy versus direct laryngoscopy for endotracheal intubation: A systematic review and meta-analysis. Canadian Journal of Anesthesia / Journal Canadien d’Anesthésie, 59, 41–52. doi:10.1007/s12630-011-9620-5 PMC3246588 22042705

[bibr16-0018720819887575] Grudin J. (2017). From tool to partner: The evolution of human-computer interaction. Synthesis Lectures on Human-Centered Interaction, 10(1), i–183.

[bibr17-0018720819887575] Grundgeiger T. Huber S. Reinhardt D. Steinisch A. Happel O. Wurmb T. (2019, May 4–9). Cognitive aids in acute care: Investigating how cognitive aids affect and support in-hospital emergency teams. Paper presented at the 2019 CHI Conference on Human Factors in Computing Systems, Glasgow, UK.

[bibr18-0018720819887575] Harbich S. Hassenzahl M. (2017). User experience in the work domain: A longitudinal field study. Interacting With Computers, 29, 306–324. doi:10.1093/iwc/iww022

[bibr19-0018720819887575] Harrison A. M. Gajic O. Pickering B. W. Herasevich V. (2016). Development and implementation of sepsis alert systems. Clinics in Chest Medicine, 37, 219–229. doi:10.1016/j.ccm.2016.01.004 27229639 PMC4884325

[bibr20-0018720819887575] Hassenzahl M. (2010). Experience design: Technology for all the right reasons. Synthesis Lectures on Human-Centered Informatics, 3(1), 1–95. doi:10.2200/S00261ED1V01Y201003HCI008

[bibr21-0018720819887575] Hassenzahl M. Platz A. Burmester M. Lehner K. (2000, April 1–6). Hedonic and ergonomic quality aspects determine a software’s appeal. Paper presented at the SIGCHI Conference on Human Factors in Computing Systems, The Hague, The Netherlands.

[bibr22-0018720819887575] Hassenzahl M. Wiklund-Engblom A. Bengs A. Hägglund S. Diefenbach S. (2015). Experience-oriented and product-oriented evaluation: Psychological need fulfillment, positive affect, and product perception. International Journal of Human-Computer Interaction, 31, 530–544. doi:10.1080/10447318.2015.1064664

[bibr23-0018720819887575] Helmreich R. L. Merritt A. C. Wilhelm J. A. (1999). The evolution of crew resource management training in commercial aviation. The International Journal of Aviation Psychology, 9, 19–32. doi:10.1207/s15327108ijap0901_2 11541445

[bibr24-0018720819887575] Hindmarsh J. Pilnick A. (2007). Knowing bodies at work: Embodiment and ephemeral teamwork in anaesthesia. Organization Studies, 28, 1395–1416. doi:10.1177/0170840607068258

[bibr25-0018720819887575] Hollnagel E. (2014). Safety-I and Safety-II: The past and future of safety management. London, England: CRC Press.

[bibr26-0018720819887575] Hollnagel E. Wears R. L. Braithwaite J. (2015). From Safety-I to Safety-II: A white paper. The resilient health care net. Odense: University of Southern Denmark; Gainesville: University of Florida; Macquarie Park, NSW: Macquarie University.

[bibr27-0018720819887575] Hornbæk K. Hertzum M. (2017). Technology acceptance and user experience: A review of the experiential component in HCI. ACM Transactions on Computer-Human Interaction (TOCHI), 24(5), Article 33.

[bibr28-0018720819887575] Hornbæk K. Oulasvirta A. (2017, May 6–11). What is interaction? Paper presented at the 2017 CHI Conference on Human Factors in Computing Systems, Denver, CO.

[bibr29-0018720819887575] Hornecker E. Marshall P. Hurtienne J. (2017, May 6–11). Locating theories of embodiment along three axes: 1st–3d person, body-context, practice-cognition. Paper presented at the Workshop Position Paper for CHI 2017 Workshop on Soma-Based Design Theory, Denver, CO.

[bibr30-0018720819887575] Huta V. Ryan R. M. (2010). Pursuing pleasure or virtue: The differential and overlapping well-being benefits of hedonic and eudaimonic motives. Journal of Happiness Studies, 11, 735–762. doi:10.1007/s10902-009-9171-4

[bibr31-0018720819887575] ISO 9241-2. (1992). Ergonomic requirements for office work with visual display terminals (VDTs)—Part 2: Guidance on task requirements.

[bibr32-0018720819887575] ISO 9241-210. (2019). Ergonomics of Human System Interaction—Part 210: Human-centred design for interactive systems.

[bibr33-0018720819887575] Jiang M. Y. Liu S. L. Feng Q. M. Gao J. Q. Zhang Q. (2018). Usability study of the user-interface of intensive care ventilators based on user test and eye-tracking signals. Medical Science Monitor, 24, 6617–6629. doi:10.12659/msm.909933 30232319 PMC6161566

[bibr34-0018720819887575] Kaptelinin V. Nardi B. (2012). Activity theory in HCI: Fundamentals and reflections. Synthesis Lectures Human-Centered Informatics, 5(1), 1–105. doi:10.2200/S00413ED1V01Y201203HCI013

[bibr35-0018720819887575] Karvonen H. Koskinen H. Haggrén J. (2012, October 14–17). Defining user experience goals for future concepts. A case study. Paper presented at the NordiCHI2012 UX Goals 2012 Workshop Proceedings, Copenhagen, Denmark.

[bibr36-0018720819887575] Karvonen H. Koskinen H. Tokkonen H. Hakulinen J. (2014). Evaluation of user experience goal fulfillment: Case remote operator station. Cham, Switzerland: Springer.

[bibr37-0018720819887575] King A. Weavind L. M. (2017). General anesthesia: Induction. Retrieved from https://www.uptodate.com/contents/general-anesthesia-induction?source=search_result&search=general%20anesthesia&selectedTitle=3~150

[bibr38-0018720819887575] Lee J. D. Wickens C. D. Liu Y. Boyle L. N. (2017). Designing for people: An introduction to human factors engineering. Charleston, SC: CreateSpace.

[bibr39-0018720819887575] Lu Y. Roto V. (2015). Evoking meaningful experiences at work—A positive design framework for work tools. Journal of Engineering Design, 26, 99–120. doi:10.1080/09544828.2015.1041461

[bibr40-0018720819887575] McCarthy J. C. O’Connor B. (1999). The context of information use in a hospital as simultaneous similarity–difference relations. Cognition, Technology & Work, 1, 25–36. doi:10.1007/s101110050008

[bibr41-0018720819887575] McCarthy J. C. Wright P. (2004). Technology as experience. Cambridge, MA: The MIT Press.

[bibr42-0018720819887575] McCarthy J. C. Wright P. (2005). Putting “felt-life” at the centre of human–computer interaction (HCI). Cognition, Technology & Work, 7, 262–271.

[bibr43-0018720819887575] Mekler E. D. Hornbæk K. (2016, May 7–12). Momentary pleasure or lasting meaning? Distinguishing eudaimonic and hedonic user experiences. Paper presented at the 2016 CHI Conference on Human Factors in Computing Systems, San Jose, CA.

[bibr44-0018720819887575] Mekler E. D. Hornbæk K. (2019, May 4–9). A framework for the experience of meaning in human-computer interaction. Paper presented at the CHI Conference on Human Factors in Computing Systems Proceedings (CHI 2019), Glasgow, UK.

[bibr45-0018720819887575] Mentler T. Herczeg M. (2016). On the role of user experience in mission- or safety-critical systems. In Mensch und Computer 2016–Workshopband. Retrieved from https://dl.gi.de/handle/20.500.12116/270

[bibr46-0018720819887575] Mewes A. Hensen B. Wacker F. Hansen C. (2017). Touchless interaction with software in interventional radiology and surgery: A systematic literature review. International Journal of Computer Assisted Radiology and Surgery, 12, 291–305. doi:10.1007/s11548-016-1480-6 27647327

[bibr47-0018720819887575] Nielsen J. (1993). Usability engineering. Fremont, CA: Morgan.

[bibr48-0018720819887575] Norros L. (2014). Developing human factors/ergonomics as a design discipline. Applied Ergonomics, 45, 61–71. doi:10.1016/j.apergo.2013.04.024 23768732

[bibr49-0018720819887575] Obrist M. Roto V. Vermeeren A. Väänänen-Vainio-Mattila K. Law E. L.-C. Kuutti K. (2012, May 5–10). In search of theoretical foundations for UX research and practice. Paper presented at the CHI’12 Extended Abstracts on Human Factors in Computing Systems, Austin, TX.

[bibr50-0018720819887575] Rogers Y. (2012). HCI theory: Classical, modern, and contemporary. Synthesis Lectures on Human-Centered Informatics, 5(2), 1–129. doi:10.2200/S00418ED1V01Y201205HCI014

[bibr51-0018720819887575] Ryan R. M. Deci E. L. (2001). On happiness and human potentials: A review of research on hedonic and eudaimonic well-being. Annual Review of Psychology, 52, 141–166. 10.1146/annurev.psych.52.1.141 11148302

[bibr52-0018720819887575] Savioja P. Liinasuo M. Koskinen H. (2014). User experience: Does it matter in complex systems? Cognition, Technology & Work, 16, 429–449. doi:10.1007/s10111-013-0271-x

[bibr53-0018720819887575] Savioja P. Norros L. (2013). Systems usability framework for evaluating tools in safety–critical work. Cognition, Technology & Work, 15, 255–275. doi:10.1007/s10111-012-0224-9

[bibr54-0018720819887575] Sheldon K. M. Elliot A. J. Kim Y. Kasser T. (2001). What is satisfying about satisfying events? Testing 10 candidate psychological needs. Journal of Personality and Social Psychology, 80, 325–339. 11220449 10.1037/0022-3514.80.2.325

[bibr55-0018720819887575] Sittig D. F. Wright A. Osheroff J. A. Middleton B. Teich J. M. Ash J. S. . . . Bates D. W. (2008). Grand challenges in clinical decision support. Journal of Biomedical Informatics, 41, 387–392. doi:10.1016/j.jbi.2007.09.003 18029232 PMC2660274

[bibr56-0018720819887575] Spaeth J. Schweizer T. Schmutz A. Buerkle H. Schumann S. (2017). Comparative usability of modern anaesthesia ventilators: A human factors study. British Journal of Anaesthesia, 119, 1000–1008. doi:10.1093/bja/aex226 29028917

[bibr57-0018720819887575] U.S. Food and Drug Administration. (2016). Applying human factors and usability engineering to medical devices—Guidance for industry and Food and Drug Administration staff. Retrieved from https://www.fda.gov/media/80481/download

[bibr58-0018720819887575] Valenta A. L. Browning M. M. Weddle T. E. Stevenson G. W. Boyd A. D. Hynes D. M. (2010, November 11–12). Physician perceptions of clinical reminders. Paper presented at the Proceedings of the 1st ACM International Health Informatics Symposium, Arlington, VA.

[bibr59-0018720819887575] van Dijk J. Hummels C . (2017, March 20–23). Designing for embodied being-in-the-world: Two cases, seven principles and one framework. Paper presented at the Eleventh International Conference on Tangible, Embedded, and Embodied Interaction, Yokohama, Japan.

[bibr60-0018720819887575] van Dijk J. van der Lugt R. Hummels C . (2014, February 16–19). Beyond distributed representation: Embodied cognition design supporting socio-sensorimotor couplings. Paper presented at the 8th International Conference on Tangible, Embedded and Embodied Interaction (TEI ‘14).

[bibr61-0018720819887575] Wu A. W. Steckelberg R. C. (2012). Medical error, incident investigation and the second victim: Doing better but feeling worse? BMJ Quality & Safety, 21, Article 267. doi:10.1136/bmjqs-2011-000605 22213379

[bibr62-0018720819887575] Yang Q. Zimmerman J. Steinfeld A. Carey L. Antaki J. F. (2016, May 7–12). Investigating the heart pump implant decision process: Opportunities for decision support tools to help. Paper presented at the Proceedings of the 2016 CHI Conference on Human Factors in Computing Systems, San Jose, CA. 10.1145/2858036.2858373 PMC5101017 27833397

[bibr63-0018720819887575] Zeiner K. M. Burmester M. Haasler K. Henschel J. Laib M. Schippert K. (2018). Designing for positive user experience in work contexts: Experience categories and their applications. Human Technology, 14(2), 141–175. doi:10.17011/ht/urn.201808103815

